# Synthetic CT generation for pelvic cases based on deep learning in multi-center datasets

**DOI:** 10.1186/s13014-024-02467-w

**Published:** 2024-07-09

**Authors:** Xianan Li, Lecheng Jia, Fengyu Lin, Fan Chai, Tao Liu, Wei Zhang, Ziquan Wei, Weiqi Xiong, Hua Li, Min Zhang, Yi Wang

**Affiliations:** 1 Peking University People’s Hospital, Beijing, China; 2 Shenzhen United Imaging Research Institute of Innovative Medical Equipment, Shenzhen, China; 3 Zhejiang Engineering Research Center for Innovation and Application of Intelligent Radiotherapy Technology, Wenzhou, China; 4 Shanghai United Imaging Healthcare Co., Ltd. Shanghai, Shanghai, China

**Keywords:** Synthetic CT, Generative adversarial nets, Contrastive learning, Multi center

## Abstract

**Background and purpose:**

To investigate the feasibility of synthesizing computed tomography (CT) images from magnetic resonance (MR) images in multi-center datasets using generative adversarial networks (GANs) for rectal cancer MR-only radiotherapy.

**Materials and methods:**

Conventional T2-weighted MR and CT images were acquired from 90 rectal cancer patients at Peking University People’s Hospital and 19 patients in public datasets. This study proposed a new model combining contrastive learning loss and consistency regularization loss to enhance the generalization of model for multi-center pelvic MRI-to-CT synthesis. The CT-to-sCT image similarity was evaluated by computing the mean absolute error (MAE), peak signal-to-noise ratio (SNRpeak), structural similarity index (SSIM) and Generalization Performance (GP). The dosimetric accuracy of synthetic CT was verified against CT-based dose distributions for the photon plan. Relative dose differences in the planning target volume and organs at risk were computed.

**Results:**

Our model presented excellent generalization with a GP of 0.911 on unseen datasets and outperformed the plain CycleGAN, where MAE decreased from 47.129 to 42.344, SNRpeak improved from 25.167 to 26.979, SSIM increased from 0.978 to 0.992. The dosimetric analysis demonstrated that most of the relative differences in dose and volume histogram (DVH) indicators between synthetic CT and real CT were less than 1%.

**Conclusion:**

The proposed model can generate accurate synthetic CT in multi-center datasets from T2w-MR images. Most dosimetric differences were within clinically acceptable criteria for photon radiotherapy, demonstrating the feasibility of an MRI-only workflow for patients with rectal cancer.

## Introduction

Recently, Magnetic resonance only (MR-only) radiotherapy has become a common research focus since it first raised, due to the superior soft-tissue contrast of MR images compared to computed tomography (CT) [1, 2]. Moreover, MR-only radiotherapy avoids additional radiation [3]. However, MR images do not contain electron density information, which is necessary for dose calculation in MR-only radiotherapy [4]. The standard solution is to generate synthetic CT (sCT) from MR images. Lately, with the development of deep learning, it has also shown great potential in the generation of sCT. Deep learning methods use large-scale image samples more efficiently to learn complex MR-to-CT mapping than conventional methods [5]. Besides, sCT can be generated during the model inference phase in just a few seconds, enabling faster model deployment [5].

The 2D Deep Convolutional Neural Network (DCNN) were first applied to the generation of sCT from MR images in the brain [5]. With the proposal of Generative Adversarial Network (GAN) [6], the application of GAN to generate images has become mainstream. For example, Conditional Generative Adversarial Network (CGAN) were used to solve the generation of sCT in the abdomen and brain [7–11]. Nevertheless, DCNN and CGAN require strictly paired data for training, which severely limits their application and increases the difficulty of data collection [12]. The CycleGAN was proposed to train the model on unpaired data through cycle consistency [13]. It was trained on unpaired data and achieved comparable results of paired data in the brain [14]. However, cycle-consistency is often too restrictive which assumes that the relationship between the two domains is a bijection in CycleGAN [15]. The contrastive learning (CL) loss was firstly utilized to enlarge the mutual information between the same location of input and synthetic images in CUT followed by some improvements, such as NEGCUT and F-LSeSim [16–18]. However, the semantic relationship between image patches is ignored and all negative image patches are considered equal probability patches.

Currently, there are some papers discussing the generalization of models in generation tasks [19–21], but a few researches focus on improving the generalization performance in multi-central datasets. This is commonly achieved by domain adaptation or using strong data augmentation in segmentation tasks [22–25]. However, domain adaptation needs to retrain the model using unseen domain data, reducing the practicability of the model [24]. Strong data augmentation, such as color augmentation, could destroy the distribution of original data and cannot be applied directly in generation tasks.

In this study, a novel framework was developed to enhance the generalization of the model in multi-central datasets using the consistency regularization learned from semi-supervised learning [26, 27]. The improved contrastive learning loss in consideration of semantic relationship was employed to enhance the structural consistency between MR and sCT.

## Materials and methods

### Data acquisition

The study cohort consisted of 90 patients diagnosed with rectal cancer from April 2018 to March 2021 at Peking University People’s Hospital (PUPH) and 19 patients diagnosed with rectal or prostate cancer in public datasets from three different Swedish radiotherapy departments [28]. Enough patient data of rectal cancer with consistent standards can be provided in this center. One of the aim of this work is to build an MR-only workflow for rectal cancer treatment. The data acquisition parameters are shown in Table 1.

**Table 1:** The dataset acquisition parameters

	PUPH	Public datasets		
		Site 1	Site 2	Site 3
Number of patients	90	8	7	4
MRI				
Manufacturer	GE Discovery MR750	GE Discovery MR750	Siemens Aera	GE Signa PET/MR
Field strength (T)	3	3	1.5	3
Spatial resolution (mm^3^)	0.67*0.67*7	0.875*0.875*3	0.875*0.875*2.5 1.1*1.1*2.5	0.875*0.875*2.5
CT				
Manufacturer	Phillips	Siemens	Toshiba	Siemens
Spatial resolution (mm^3^)	1*1*3	0.98*0.98*3	1*1*2	0.98*0.98*2.5

The age distribution of patients was 43–83 years in PUPH cohort. CT scanning was performed with a Philips 16-row large-aperture analog positioning machine with a flat table top. Scan parameters: 140 kV, 280 mAs, layer thickness 3 mm. MRI of the pelvis was performed using a GE Discovery MR750 3.0T MR scanner with the curved table top. The scanning sequence and parameters are as follows: High-resolution non-fat-suppressed fast recovery fast spin-echo (FRFSE) T2-weighted imaging sequence, TR 3 200 ms, TE 85 ms, slice thickness 3 mm, slice interval 0.5 mm, a field of view 32 cm×26 cm.

T2-weighted MR and CT data were collected for 19 patients at three different sites in public datasets. All patients were scanned using a coil setup that not affects the outline of the patient in the radiotherapy treatment position with a flat table top.

### Preprocessing

External contours of CT and MR images were generated in Treatment Planning System (TPS) (UIH, Shanghai United Imaging Healthcare Co., Ltd.). All CT and MR voxels outside the external contour were assigned to intensity of -1024 and 0, respectively. The intensities of CT images were linearly mapped from [-1024; 1500] to [-1; 1]. The intensities of MR images were clipped beyond the 95th percentile, and then the intensities were also linearly mapped to [-1; 1]. Deformable registration was performed on MR and CT images by NiftyReg open-source software [29], and the registration results were revised by an experienced physician.

The three-fold cross-validation was used in this study. The specific division of data is as follows: 30 cases were randomly selected from PUPH cohort and one center was picked from public datasets as test datasets and the rest data served as train datasets in each fold.

### Network architectures

**Fig. 1:**
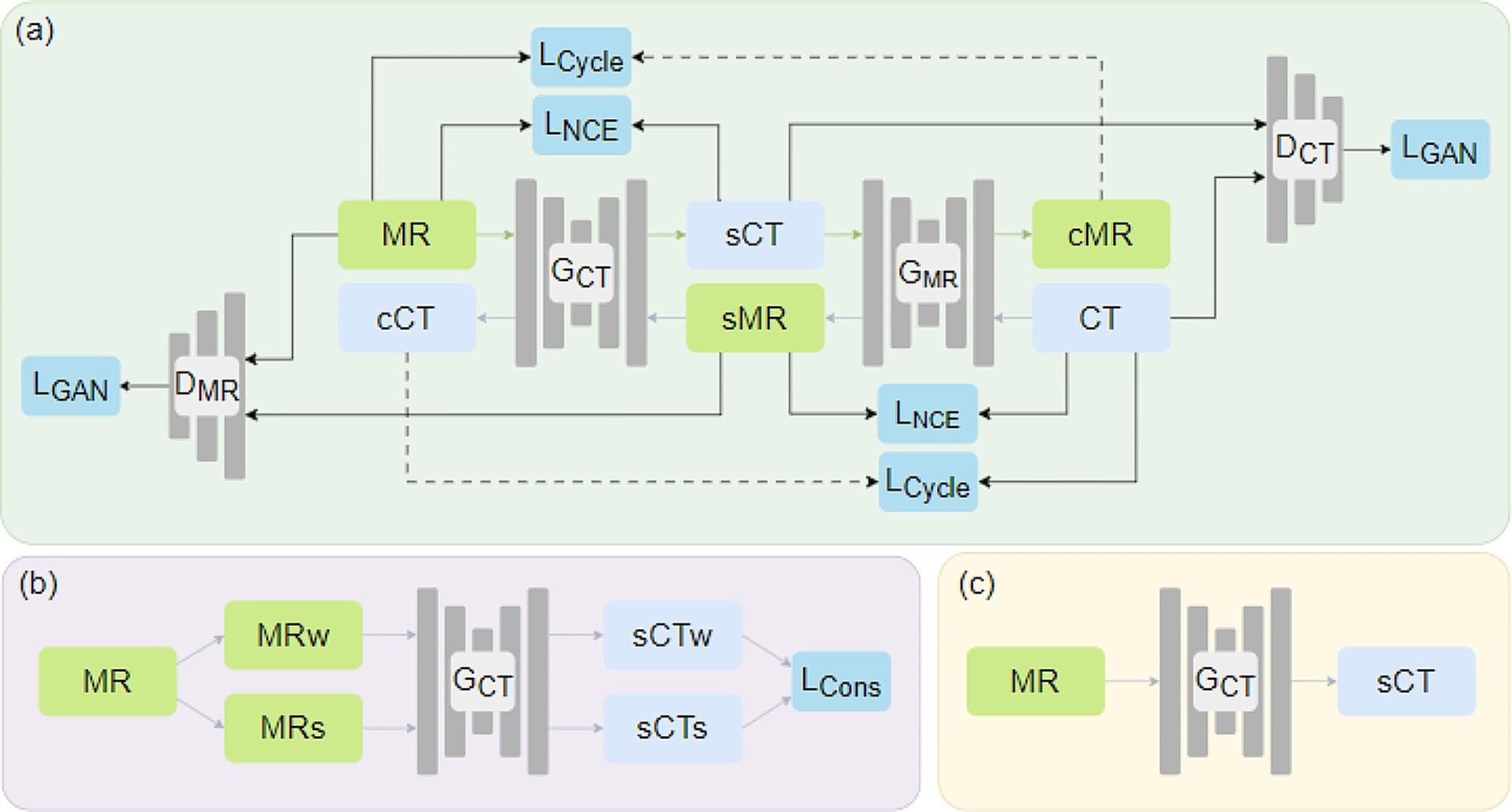
Illustration of architecture of CRGAN. (**a**) and (**b**) are the training phase of CRGAN, (**c**) is the inference phase of CRGAN

As shown in Fig. 1(a), the proposed CRGAN (Consistency Regularization Generative Adversarial Network) contained two generators and discriminators. Wherein the generator G_CT_ provides MR to CT mapping, the generator G_MR_ provides CT to MR mapping. Furthermore, the discriminator D_MR_ and D_CT_ were used to distinguish between real images and synthetic images [13].

Figure 1a) and b) show the training phase of CRGAN. In order to improve the generalization of the model, consistency regularization similar to Flexmatch was employed to optimize the G_CT_ [26], as shown in Fig. 1b). The weak and strong data augmentation was performed in the same MR image to obtain the MRw and MRs. In weak data augmentation, operations such as like flipping along the vertical direction, scaling and clip to certain size, random clip and resize, and rotation with random degree between 0 ~ 360° were applied without changing the value distribution of the image. In strong data augmentation, MR images were further operated with color augmentation, includes the methods that will change the voxel values of images, such as altering the brightness using gamma changes, applying Gaussian filtering to the image. Then the consistency regularization loss was added to ensure that the weak and strong augmentation MR images would generate similar sCT. Figure 1c) show the inference phase of the model.

A 2.5D image was token as input of CRGAN, which contains 3 adjacent layers is extract from a 3D image. The ADAM optimization was used to minimize the loss function [30]. CRGAN was initialized using the He_normal initialization method [31].

#### Generator

**Fig. 2:**
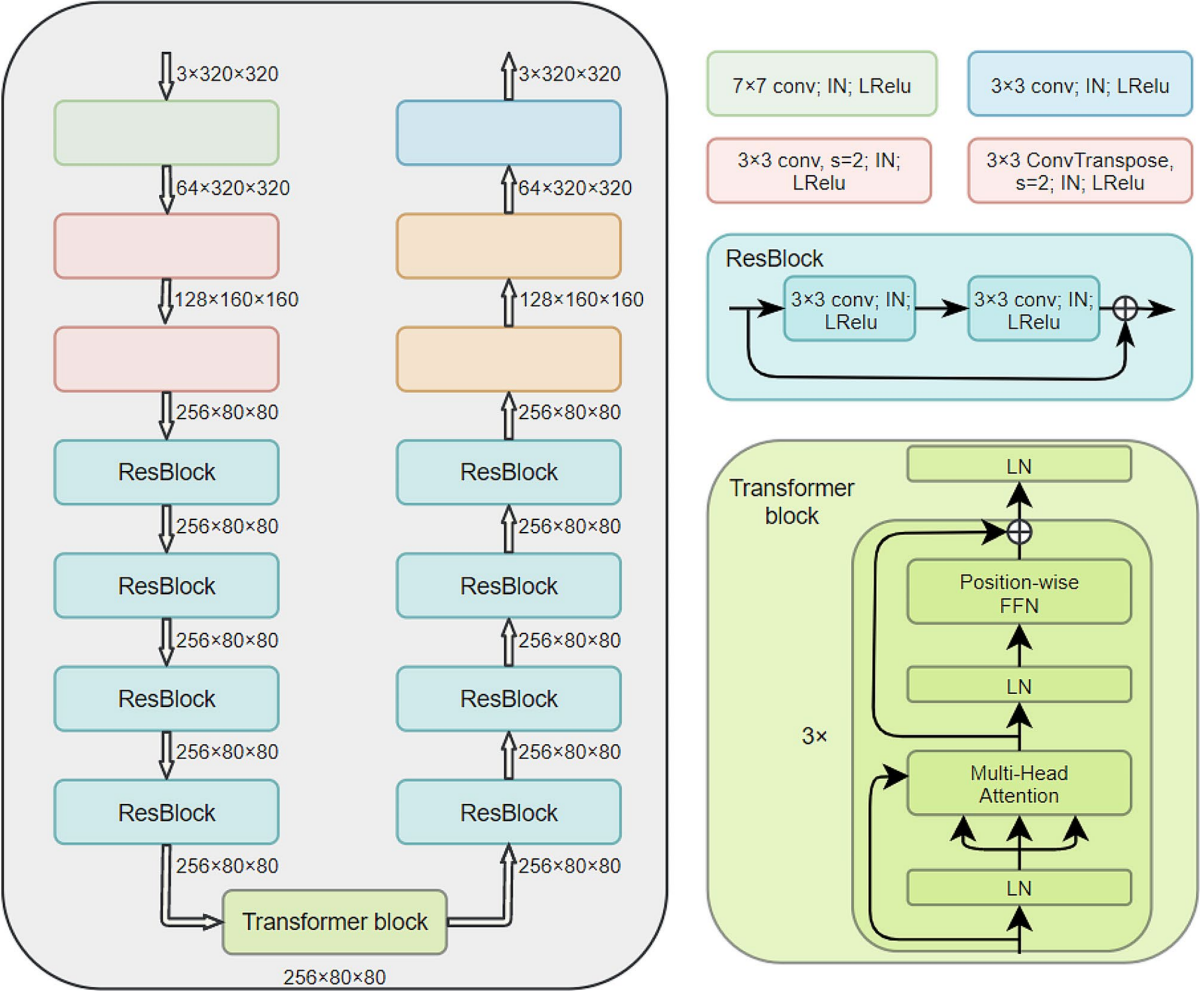
Illustration of architecture of generator of CRGAN. IN: Instance Norm, LRelu: LeakyRelu, LN: Layer norm, FFN: Feed Forward Network

The Transformer module was employed in the generator of CRGAN, as shown in Fig. 2. The Transformer module can pay attention to the global connection of features compared with the convolution module [32]. There are tremendous work including imaging segmentation and translation adopting transformer structures and obtain promising performance. It is generally believed that the Transformer module is more effective than the convolution module in extracting deep features [33], so we put the Transformer module on the last layer of the encoder of the generator.

#### Discriminator

All the discriminator networks in CRGAN shared the same architecture, obtained by spectral normalization of the discriminator in the plain CycleGAN [13]. Spectral normalization introduces regularity constraints from the perspective of the spectral norm of the parameter matrix of each layer of the neural network [34], so that the neural network has better insensitivity to input disturbances, thus making the training process more stable and easier to converge.

#### Loss function

In this study, a mixed loss function including adversarial loss, cycle consistency loss, consistency regularization loss and contrastive learning loss was used as the objective function, which is defined as follows:

$$\begin{aligned} Loss&={L}_{adv}+{L}_{cycle}+{L}_{consistency regulation}\\ &\quad +{L}_{contrastive learning}\end{aligned}$$

The adversarial loss (shown as L_GAN_ in Fig. 1) function optimized the generator and discriminator. For the generator G_CT_ and its discriminator D_CT_, the adversarial loss function is defined as

$${L}_{adv}\left({G}_{CT}, {D}_{CT}\right)={D}_{CT}\left({G}_{CT}\left({I}_{MR}\right)\right)+(1-{D}_{CT}({I}_{CT}\left)\right)$$

Where I_CT_ and I_MR_ represent unpaired input CT and MR images. In the training phase, G_CT_ generates a synthetic CT image G_CT_(I_MR_) that is close to the real CT image, while D_CT_ is to distinguish the synthetic CT image G_CT_(I_MR_) from a real image I_CT_. Likewise, the adversarial loss functions for G_MR_ and D_MR_ are defined as

$${L}_{adv}\left({G}_{MR}, {D}_{MR}\right)={D}_{MR}\left({G}_{MR}\left({I}_{CT}\right)\right)+(1-{D}_{MR}({I}_{MR}\left)\right)$$

The cycle-consistent loss function optimized the G_CT_ and G_MR_, forcing the reconstructed images G_CT_ (G_MR_(I_CT_)) and G_MR_ (G_CT_(I_MR_)) to be the same as their input I_CT_ and I_MR_. This loss function is defined as

$$\begin{aligned} {L}_{cycle}\left({G}_{CT}, {G}_{MR}\right)&=\Vert{G}_{CT}\left({G}_{MR}\left({I}_{CT}\right)\right)-{I}_{CT}\Vert\\ &\quad+\Vert{G}_{MR}\left({G}_{CT}\left({I}_{MR}\right)\right)-{I}_{MR}\Vert\end{aligned}$$

The consistency regularization loss (shown as L_Cycle_ in Fig. 1. (a)) function optimized the G_CT_, ensuring MR images enhanced by weak and strong augmentation would generate similar sCT. This loss function is defined as

$${L}_{consistency regularization}\left({G}_{CT}\right)=\Vert{G}_{CT}\left({I}_{MRw}\right)-{G}_{CT}\left({I}_{MRs}\right)\Vert$$

#### Contrastive learning loss

The CL loss (shown as L_Cons_ in Fig. 1. (b)) optimized the generator G_CT_ and G_MR_. The semantic relation consistency (SRC) regularization with the decoupled contrastive learning was used [15]. SRC utilizes the semantics feature by focusing on the semantic relation between the image patches from a single image. In addition, the hard negative mining strategy is explored by exploiting the semantic relation [15]. This loss function is defined as

$$\begin{aligned}{L}_{CL}&={\gamma }_{SRC}\sum _{k=1}^{K}JSD\left(\frac{\text{exp}\left({z}_{k}^{T}{z}_{i}\right)}{{\sum }_{j=1}^{K}\text{exp}\left({z}_{k}^{T}{z}_{j}\right)}\right|\left|\frac{\text{exp}\left({w}_{k}^{T}{w}_{i}\right)}{{\sum }_{j=1}^{K}\text{exp}\left({w}_{k}^{T}{w}_{j}\right)}\right)\\ &+{\gamma }_{hDCE}{\mathbb{E}}_{\left(z,w\right)\sim{p}_{ZW}}[-log\frac{\text{e}\text{x}\text{p}({w}^{T}z/\tau )}{N{\mathbb{E}}_{{z}^{-}\sim{q}_{{Z}^{-}}}[\text{e}\text{x}\text{p}({w}^{T}{z}^{-}/\tau \left)\right]}] \end{aligned}$$

where ${\gamma }_{SRC}$ and ${\gamma }_{hDCE}$ are weighting parameters; JSD represents Jensen-Shannon Divergence; z_k_ and z_i_ are the corresponding embedding vectors of k-th location and i-th location patches of the input image; w_k_ and w_i_ are the corresponding embedding vectors of k-th location and i-th location patches of the synthetic image; the negative sampling is modeled by von Mises-Fisher distribution:

$${z}^{-}\sim{\text{q}}_{{Z}^{-}}({z}^{-};z, \gamma )=\frac{1}{{N}_{q}}\text{e}\text{x}\text{p}\left\{\gamma \right({z}^{T}{z}^{-}\left)\right\}{p}_{Z}\left({z}^{-}\right)$$

where N_q_ is a normalization constant;γ is a hyper-parameter determining the hardness of the negative samples.

### Evaluation metrics

Referring to the main stream articles of imaging translation, MAE, SNRpeak and SSIM are three commonly used metrics to measure the quality of images. MAE evaluates the voxel-vise similarity between images, SNRpeak evaluates the image quality, and the SSIM evaluates the structure similarity between two images. To evaluate the generalization performance across multi-center data, we proposed a new metric GP (Generalization Performance) based on the above metrics. To evaluating the similarity between a sCT image and real CT, their image quality, HU values, and anatomical structures similarity are most important aspects, and the metrics mentioned above can evaluate the performance from these aspects.

#### MAE (mean absolute error)

MAE can be used to evaluate the difference in HU values between sCT and CT images as follows:

$$\text{M}\text{A}\text{E}=\frac{1}{N}\sum _{i=1}^{N}\left|{CT}_{i}-{sCT}_{i}\right|$$

Where the index *i* represents each voxel of the image.

#### SNRpeak (Peak Signal-to-noise ratio)

**SNRpeak** provides an objective measure of image distortion or noise level, as follows:

$$\text{S}\text{N}\text{R}\text{p}\text{e}\text{a}\text{k}=10\text{*}{log}_{10}\left(\frac{{MAX}_{I}^{2}}{MSE}\right)$$

#### SSIM (Structural Similarity index)

SSIM analyzes the similarity between images in terms of brightness, contrast, and structure, as follows: C1 and C2 are constants. 

$$\text{S}\text{S}\text{I}\text{M}=\frac{(2{\mu }_{x}{\mu }_{y}+{C}_{1})(2{\sigma }_{xy}+{C}_{2})}{({{\mu }_{x}}^{2}+{{\mu }_{y}}^{2}+{C}_{1})({{\sigma }_{x}}^{2}+{{\sigma }_{y}}^{2}+{C}_{2})}$$

#### Model’s generalization analysis

We proposed a new metric GP (Generalization Performance) to assess the generalization of model on unseen datasets. It is computed as follows:

$$\text{G}\text{P}=\frac{{MAE}_{seen}}{{MAE}_{unseen}}*\frac{{PSNR}_{unseen}}{{PSNR}_{seen}}*\frac{{SSIM}_{unseen}}{{SSIM}_{seen}}$$

GP is composed of three parts, each of which reflects the model’s generalization in MAE, SNRpeak and SSIM, respectively. Therefore, this indicator can comprehensively reflect the generalization performance on unseen datasets. The seen and unseen datasets are defined as the datasets trained on and test on, respectively. In such definition, seen and unseen datasets are whole datasets, not the training or testing splits from one dataset or mixed multi-datasets. The larger the indicator is, the better the generalization performance is. When the indicator value is close to 1, it indicates that the performance of the model on unseen datasets is equal to that on the seen datasets, indicating excellent model generalization.

#### Dosimetric analysis

Dosimetric accuracy of sCT images were evaluated by clinical rectal cancer treatment planning. A dose of 5000 cGy was prescribed for the primary tumor target and the photon plan was designed for each test data using real CT images (TPS, UIH). Then the segmentation and plan of real CT image were copied to the sCT image. The dose distribution of the plan generated on real CT was recalculated on the sCT to investigate the gap between them. The dose matrix has a resolution of 3 × 3 × 3 mm^3^ and covers the main region of interest (ROI).

## Results

### Image comparison

**Fig. 3:**
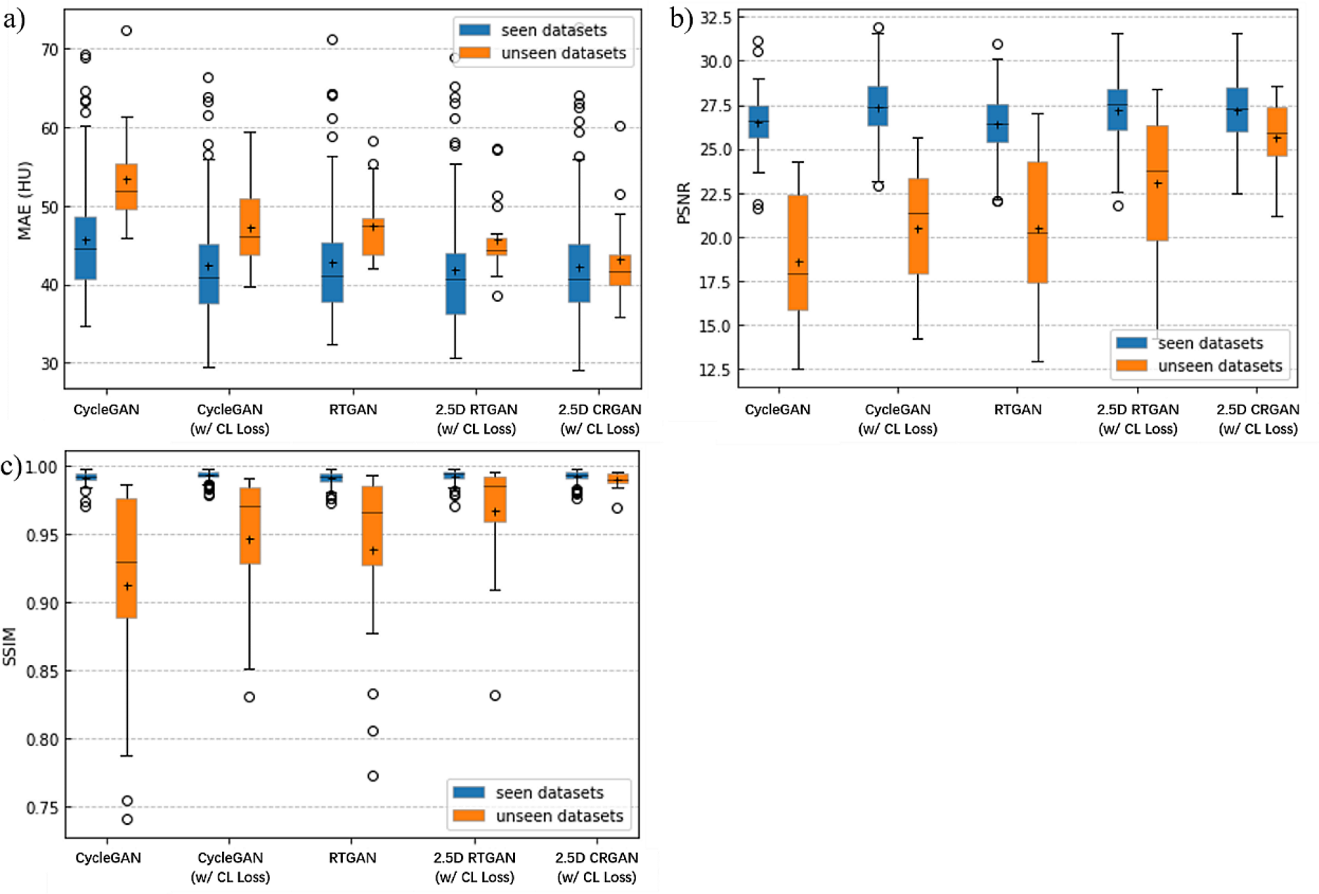
Performance of different models on the seen and unseen datasets

The results of the two samples are shown in Fig. 3. The first column shows the input MR image and its highlighted region. The second to the sixth columns show the real CT images, the prediction results of CycleGAN, CycleGAN with CL loss, RTGAN (ResTransformer Generative Adversarial Network), 2.5D RTGAN with CL loss and 2.5D CRGAN with CL loss. The first sample was selected from validation sets of PKPU that can be considered as the seen data since the train set included some other data from PKPU. The second sample was picked from public datasets regarded as unseen data for this whole center data were taken as validation datasets.

**Fig. 4:**
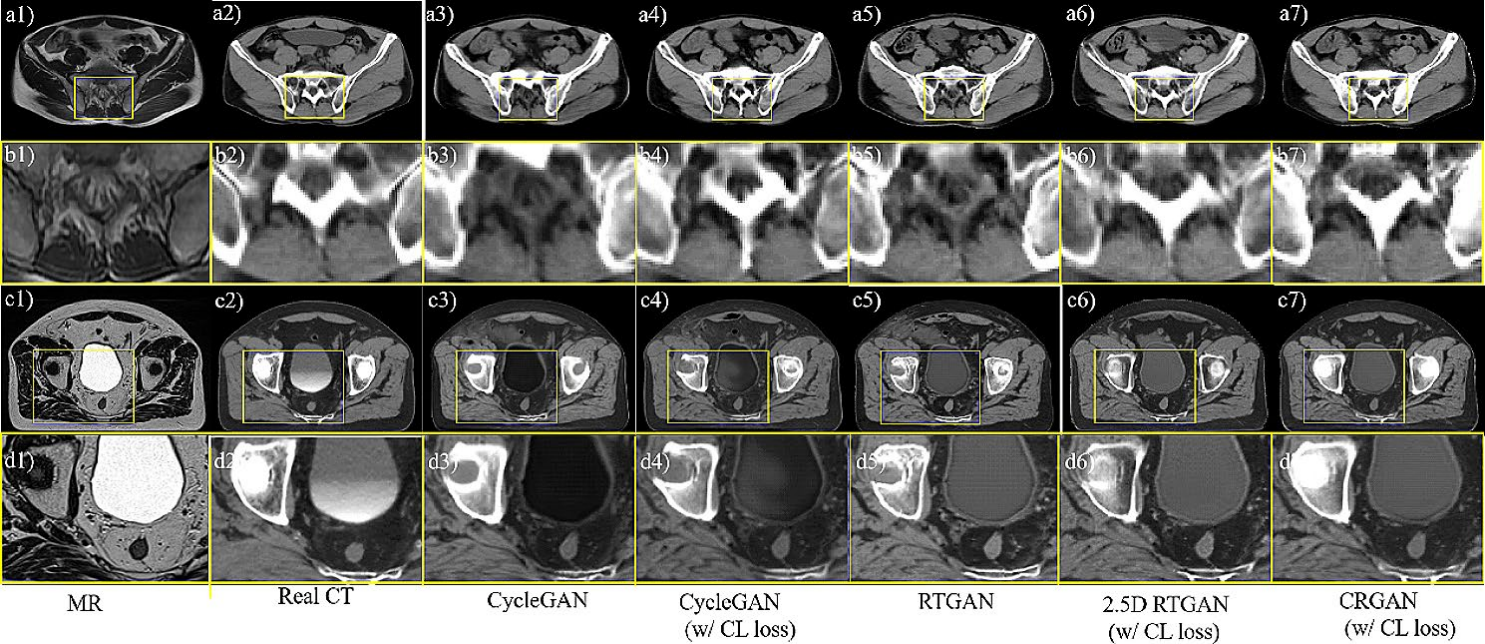
sCT images generated by different model. The first and third rows show real MR (a1, c1), real CT (a2, c2) and the sCT images generated by CycleGAN (a3, c3), CycleGAN with CL loss (a4, c4), RTGAN (a5, c5), 2.5D RTGAN with CL loss (a6, c6) and our model (a7, c7). The second and fourth rows highlight the ROIs outlined by the yellow box on each corresponding image

SCT generated by each model on the seen data were accepted in most areas, with good contrast between the bone and their surrounding soft tissues in the first sample. However, there were some mismatches appearing in the local bone region in sCT, as shown in Fig. 4b3) and b5). By adding the CL loss to the model, the edge of bone can be improved in Fig. 4b4) and b6). For the second sample in the unseen dataset, our proposed model (Fig. 4(d7)) presented more accurate shape and values of bone than that of other models (Fig. 4(d3) ~ Fig. 4(d6)) by adding the consistency regularization.

To evaluate the accuracy of sCT images generated for treatment planning, we calculated the MAE, SSIM and SNRpeak for the entire outer contour of each patient in the test set under each model, as shown in Table 2. As can be seen from Table 2, adding the Transformer module or using CL loss could improve the result of sCT. The performance of sCT could be further enhanced by combining both of them into CycleGAN. However, the GP values of the above models were relatively low, and the generalization performance of the model was greatly enhanced after using the CL loss.

**Table 2:** Comparison of MAE, SSIM and SNRpeak of sCT produced by different methods in the whole pelvic region

Method	MAE↓	SNRpeak↑	SSIM↑	GP↑
CycleGAN	47.129	25.167	0.978	0.545
CycleGAN (w/ CL loss)	43.753	26.211	0.985	0.645
RTGAN	43.704	25.344	0.982	0.66
2.5D RTGAN (w/ CL loss)	42.557	26.268	0.986	0.690
2.5D CRGAN (w/ CL loss)	42.344	26.979	0.992	0.911

The training time of our model with the training dataset is 6 days, and the inference time is 3s per image due to the sliding window strategy on a Nvidia 3090. As a comparison, CycleGAN used 5 days of training time and 2.4 s of inference time with the same equipment.

### Model generalization

The Fig. 4 and Table 2 have initially shown us the generalization of different models in section “Image comparison”. Figure 3 shows the MAE, SNRpeak and SSIM of different models on the seen and unseen datasets. There was big gap in performance between seen and unseen datasets before adopting consistency regularization. This gap could be well compensated after using consistency regularization in our proposed model.

### CL loss

Table 3 shows the effect of CL loss addition on MAE of different models. We reported the MAE of the main organs in abdomen, including bladder, rectum, and femur heads. The performance of sCT was enhanced in both bone and soft tissue regions by the introduction of CL loss. The result of main organs was shown in Table 3. In addition, there was a greater decrease in the MAE in bone region, which is consistent with the phenomenon observed in Fig. 4.

**Table 3:** Comparison of MAE on the soft tissue and bone region for the sCT generated through different methods

Region	CycleGAN	CycleGAN (w/ CL loss)	RTGAN	2.5D RTGAN (w/ CL loss)	2.5D CRGAN (w/o CL loss)	2.5D CRGAN (w/ CL loss)
Bladder	61.45	47.92	48.23	52.67	39.82	35.84
Rectum	59.66	47.02	45.45	42.52	42.12	40.44
Femur_Head_L	163.02	157.23	140.23	121.17	134.56	123.75
Femur_Head_R	181.73	163.39	150.34	132.60	140.28	131.89

### Dose comparison and Gamma index

For each patient, a photon plan using Volumetric Modulated Arc Therapy (VMAT) was generated and the DVH was analyzed for target and critical structures. DVH parameters such as mean dose (Dmean), maximum dose (Dmax), D95%, and D50% were calculated for Planning Target Volume (PTV), Clinical Target Volume (CTV), bladder, left femur head and right femur head. The prescription dose was 50 Gy and 2 Gy/fraction for treatment.

**Fig. 5:**
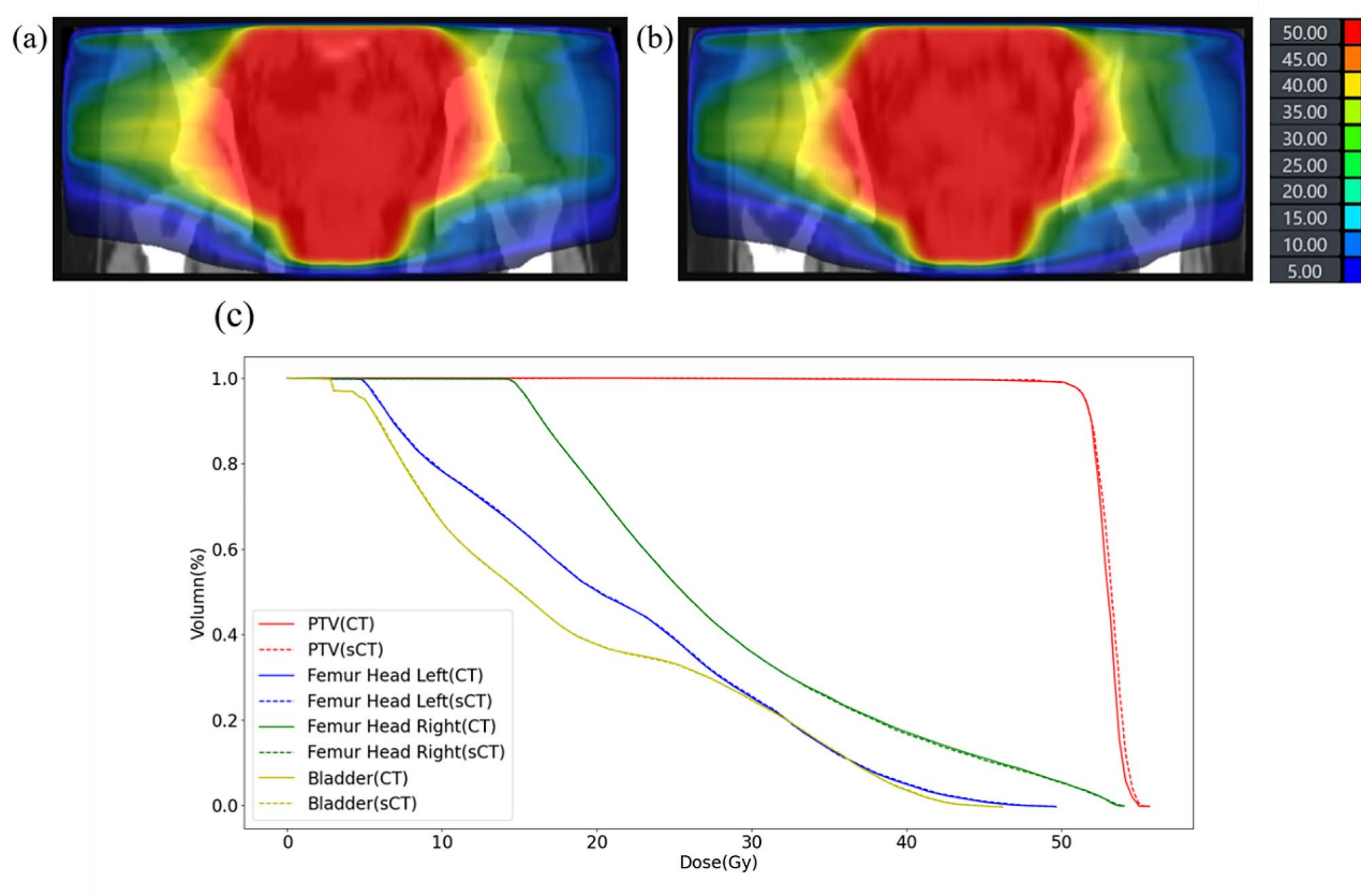
(a) dose distribution map in CT. (b) dose distribution map in synthetic CT. (c) DVH plot with corresponding PTV and OARs

Figure 5 and Table  4 show the comparison of DVH obtained from the radiation planning dose calculation between real CT and sCT generated by 2.5D CRGAN (w/ CL loss). It can be seen that most of the relative differences in the DVH indicators were less than 1%, indicating that the current sCT can meet the needs of radiotherapy planning.

**Table 4:** Relative dose differences between sCT and CT plans. >0.05: not significant, paired two tailed t-test

DVH indicator		CT vs. synthetic CT mean relative differences(range)	*p*-value
PTV Rectum			
	D_mean_(100%)	0.848(-1.880 to 1.970)	0.208
	D_max_(100%)	0.119(-1.130 to 2.161)	0.866
	D_95_(100%)	1.255(0.0200 to 2.492)	0.972
	D_50_(100%)	0.945(-0.890 to 1.956)	0.155
CTV Rectum			
	D_mean_(100%)	0.861(-1.550 to 1.799)	0.486
	D_max_(100%)	0.116(-1.130 to 2.288)	0.871
	D_95_(100%)	1.580(0.039 to 2.854)	0.882
	D_50_(100%)	0.918(-0.860 to 1.871)	0.185
Bladder			
	D_mean_(100%)	0.830(-1.520 to 4.751)	0.839
	D_max_(100%)	1.149(-0.360 to 2.439)	0.236
	D_95_(100%)	0.677(-3.060 to 1.687)	0.922
	D_50_(100%)	0.926(-1.680 to 3.295)	0.874
Left Femur Head			
	D_mean_(100%)	-0.070(-1.110 to 0.871)	0.340
	D_max_(100%)	0.016(-1.610 to 1.626)	0.334
	D_95_(100%)	0.260(-1.890 to 1.250)	0.628
	D_50_(100%)	0.800(-1.140 to 1.180)	0.330
Right Femur Head			
	D_mean_(100%)	-0.090(-0.750 to 0.530)	0.980
	D_max_(100%)	-0.180(-3.400 to 1.395)	0.968
	D_95_(100%)	-0.180(-1.730 to 3.600)	0.990
	D_50_(100%)	-0.250(-1.350 to 1.120)	0.958

We also reported the Gamma index [35] as Table 5 to show that the results produced by our results have sufficient Gamma index for clinical use. The gamma indices (3 mm, 3%) have been calculated between three-dimension dose distribution of real CT and those of fake CT generated by proposed methods.

**Table 5:** Relative dose differences between sCT and CT plans. >0.05: not significant, paired two tailed t-test

Region	CycleGAN	CycleGAN (w/CLloss)	RTGAN	2.5DRTGAN (w/ CLloss)	2.5DCRGAN (w/ CL loss)
External	0.893	0.897	0.903	0.905	0.907
Bladder	0.902	0.852	0.848	0.903	0.951
Femur_Heads	0.903	0.915	0.922	0.874	0.924
CTV_Rectum	1.000	1.000	1.000	1.000	1.000
PTV_Rectum	0.997	0.996	0.996	0.995	0.999

## Discussion

In this study, we proposed a new model combining contrastive loss and consistency regularization for pelvic MRI-to-CT synthesis. MR images used in our model were single T2 sequences, as suggested in the previous study [36]. The experiment results in Table 2 reveal our superior performance. Primarily, our model presented excellent generalization and performed better than plain CycleGAN, where MAE decreased from 47.129 to 42.344, SNRpeak improved from 25.167 to 26.979, SSIM increased from 0.978 to 0.992 and GP increased from 0.545 to 0.911. Meanwhile, most of the relative differences in the DVH indicators are less than 1%, which is generally considered clinically acceptable. This level of accuracy suggests that the sCT provides a reliable estimate of the actual radiation exposure received by the patient’s tissues.

There are many algorithms have been employed in segmentation tasks to improve the accuracy of segmentation on unseen datasets [23–25], and some of them have obtained similar segmentation results on unseen datasets as on seen datasets [24]. However, it is not required to maintain the value distribution of the original data in the segmentation task. In order to increase the generalization performance of a model, strong data augmentation, such as color augmentation, is often used, which disrupts the value distribution of the input image. For the generation task, since the learning target is the image itself, the value distribution of the image needs to be maintained during the training process, and strong augmentation is difficult to be applied directly. Here we adopted consistency regularization similar to semi-supervised learning [26]. Weak augmentation was used to maintain the value distribution of MR data during the training of CRGAN and Strong augmentation was used to improve the generalization performance of the model. Consistency regularization was used to ensure that MR images undergoing strong augmentation and weak augmentation generate similar sCT. The generalization of 2.5D RTGAN with CL loss was poor with a GI around 0.7 and it can be improved considerably to 0.91 in our model using consistency regularization. This can also be seen from the Fig. 4 that the performance of our model on the unseen dataset was close to the performance on the seen dataset. These demonstrate the effectiveness of using strong data augmentation as well as consistency regularization.

Contrastive learning loss has been shown to be effective in the generation task [16–18]. In this study, the semantic relation was introduced into contrastive learning and the hard negative mining strategy was explored based on semantic relation. The semantic relation can enhance the structural consistency of MR and corresponding sCT image blocks. The shape of bone of sCT was significantly improved by adding contrastive learning loss, as shown in Fig. 4. It can also be seen from Table 3 that using contrastive learning can effectively improve the results of sCT, and the improvement is mainly concentrated in the bone region. These show that contrastive learning can enhance the structural consistency of MR and sCT and improve the results of sCT.

In this study, we also embedded the Transformer block into the generator to improve the performance of the model. The Transformer module was placed at the last layer of the encoder to extract deep features more efficiently. Compared with the original CycleGAN, all the performance was improved after adding the Transformer module, indicating the superiority of the Transformer in extracting deep features, which is consistent with the previous studies [33].

The existing clinical workflow can be improved and accelerated the efficiency of initial treatment. For TPS manufacturers, providing an initial model that can be used for multiple centers is very important. It can be used to verify whether the entire treatment workflow can go successfully. After training with specific center data, specialized optimization can be carried out for the center to accelerate the implementation of MR-only workflow.

This study has a few limitations. First, the model’s generalization depends heavily on whether strong augmentation can simulate the unseen dataset better. However, Clinical data are usually complex and it is challenging to simulate all clinical data through strong augmentation. The registration results of paired training data are also important, more supervise from experienced physicians is required. Therefore, the next step is to improve the generalization of the model with the help of a small number of unseen datasets. More data with standard consistence are also planned to collect in the future for better performance.

MR equipment with higher magnetic field intensity can provide higher signal-to-noise ratio, which means that when converted to pseudo-CT, the image quality will be better and the details will be clearer. High field MR can provide stronger contrast, making the differentiation between different tissues more prominent when converted to pseudo-CT, which helps in the development of diagnosis and treatment plans. The field of MR data used in our work is 1.5 T, which may limit the image quality and further in the practical clinical workflow. Finally, this study has explored little about the Transformer module and its inherent mechanism.

## Conclusion

In this study, we proposed a new model combining contrastive learning loss and consistency regularization loss for multi-center pelvic MRI-to-CT synthesis. The proposed model used a hybrid CNN and Transformer module as a generator. Our model presented excellent generalization in multi-center datasets. With an application in the pelvic region, in which MRI-CT registration is particularly hard, this method is promising for radiotherapy treatment planning and would ease the clinical workflow whilst potentially improving its accuracy.

## Data Availability

The public datasets are available from https://zenodo.org/record/583096.The datasets from PUPH are not publicly available due to privacy of patients. You can contact the corresponding author for the usage of the datasets.

## Abbreviations

CT: Computed tomography
MR: Magnetic resonance
GAN: Generative adversarial network
MAE: Mean absolute error
SNRpeak: Peak signal-to-noise ratio
SSIM: Structural similarity index
GP: Generalization Performance
DVH: Dose and volume histogram
DDCN: Deep convolutional neural networks
CGAN: Conditional generative adversarial network
CL: Contrastive learning
PUPH: Peking University People’s Hospital
FRFSE: Fast recovery fast spin-echo
TPS: Treatment planning system
SRC: Semantic relation consistency
JSD: Jensen-Shannon Divergence
RTGAN: ResTransformer Generative Adversarial Network
VMAT: Volumetric Modulated Arc Therapy
Dmean: Mean dose
Dmax: Maximum dose
CNN: Convolutional neural network
PTV: Planning Target Volume
CTV: Clinical Target Volume
